# Mechanobiological and neuromuscular responses to foot-position variations during front and back squat exercises

**DOI:** 10.3389/fphys.2025.1727141

**Published:** 2026-01-07

**Authors:** Ömer Bozkurt, Deniz Erdağ, Cevdet Tinazci, Nazım Burgul, Hasan Bekiroğulları

**Affiliations:** 1 Department of Coaching Education, Faculty of Sports Sciences, Near East University, Nicosia, Cyprus; 2 Department of Sports Science, Final International University, Mersin, Türkiye

**Keywords:** biomechanics, electromyography, foot position, kinematics, mechanobiology, muscle activation, neuromuscular adaptation, squat

## Abstract

**Introduction:**

This study investigated the mechanobiological and neuromuscular effects of three foot-position techniques—flat heel (FH), heel-elevated (HE), and forefoot-elevated (FE)—during front and back squat exercises. Variations in foot positioning were expected to influence muscle activation, joint kinematics, ground reaction forces, and postural stability.

**Methods:**

Twelve resistance-trained male athletes (mean age 23.4 ± 3.8 years; training experience 8.1 ± 2.7 years) performed squats at 70% of their one-repetition maximum (1RM) under each foot-position condition. Surface electromyography (EMG) assessed lower-limb muscle activation, while joint angles and ground reaction forces were collected through synchronized motion-capture and force-platform analyses. Measures included EMG amplitude, joint kinematics, ground reaction forces, and center-of-pressure (COP) displacement.

**Results:**

Quadriceps muscles (vastus lateralis, vastus medialis, rectus femoris) showed significantly greater activation in FH and HE compared with FE (p < 0.001), particularly during the ascent phase. Heel-elevated increased ankle dorsiflexion and squat depth, whereas FE reduced vertical ground reaction forces and shifted mechanical loading toward the posterior chain. No significant differences were observed in COPx or COPy across conditions.

**Discussion:**

Foot-position variations meaningfully altered mechanical load distribution and neuromuscular activation patterns, demonstrating human-scale mechanotransduction. The findings suggest that technique selection should be tailored to individual mobility, joint mechanics, and performance goals to optimize training outcomes and reduce injury risk.

## 1 Introduction

Strength is recognized as one of the fundamental components of athletic performance, and lower-limb strength plays a critical role not only in athletic success but also in postural control and injury prevention (Sánchez Pastor et al., 2023; Delfa-de-la-Morena et al., 2024; Schoenfeld, 2010a; Kraemer et al., 1995). Resistance training enhances performance through both muscular hypertrophy and neuromuscular adaptations that collectively improve force generation and coordination (Kraemer, 2004; Currier et al., 2023; Suchomel et al., 2016). In this context, the squat is considered one of the most frequently used multi-joint exercises targeting the lower-limb musculature, with extensive research emphasizing its central role in muscle activation, strength development, and motor control (Clark et al., 2012; van den Tillaar et al., 2020; Escamilla, 2001). Among elite strength athletes, particularly powerlifters, the squat serves as a cornerstone movement for developing maximal lower-body strength and technical efficiency (Swinton et al., 2009). Recent evidence has also shown that squat-based exercises elicit substantial gluteal hypertrophy comparable to other lower-body movements such as the hip thrust (Plotkin et al., 2023), reinforcing the squat’s mechanobiological relevance in promoting muscle adaptation through repetitive mechanical loading.

Squat variations produce distinct effects on movement mechanics, reflecting the complex interplay between joint loading strategies and the distribution of mechanical forces. While the front squat, characterized by a more upright trunk position, enhances quadriceps loading, the forward torso inclination in the back squat increases the contribution of the hamstrings, gluteus maximus, and erector spinae to trunk stabilization (Gullett et al., 2009; Coratella et al., 2021; Caterisano et al., 2002; Sinclair et al., 2016; Saeterbakken and Fimland, 2012). Changes in foot positioning further alter mechanical stress distribution and postural equilibrium during the squat. The flat-heel position supports balance (Straub and Powers, 2024; Murray et al., 2013), whereas heel-elevated facilitates ankle dorsiflexion, improves trunk alignment, and increases squat depth, thereby elevating quadriceps loading (da Costa et al., 2021; Cabral et al., 2023). Conversely, elevating the forefoot shifts the center of mass posteriorly, modifying anterior–posterior ground-reaction-force distribution and altering balance strategies, while increasing posterior-chain demand and reducing quadriceps loading (Straub and Powers, 2024; Swinton et al., 2012).

Electromyography (EMG) is widely used to assess neuromuscular responses to the mechanical variations imposed by different squat techniques. During squatting, the quadriceps function as the primary agonists, whereas the hamstrings act as synergists and stabilizers (Isear et al., 1997; Torres et al., 2020; Kubo et al., 2019). Heel-elevated has been shown to significantly increase activity of the vastus medialis obliquus (Charlton et al., 2017). From a neuromechanical perspective, variations in ankle, knee, and hip joint kinematics influence force distribution and muscle-activation patterns (Sinclair et al., 2016; Cabral et al., 2023; Monteiro et al., 2022; Goršič et al., 2024; Cotter et al., 2013). Kinetic analyses further indicate that front squats increase knee-joint moments, whereas back squats shift loading toward the hip (Sayers et al., 2020; Glassbrook et al., 2017). Collectively, electromyography activity, joint-angle behavior, and balance-related force adjustments reflect coordinated neuromuscular strategies that respond dynamically to changes in foot posture.

However, studies that comprehensively integrate electromyography, kinematic, and kinetic assessments of different squat variations are still limited. In particular, the absence of systematic comparisons among the flat-foot position, the dorsiflexion-enhanced (heel-elevated) position, and the plantar-flexion-enhanced (forefoot-elevated) position in both front and back squat styles represents a significant research gap. Addressing this gap provides a valuable mechanobiological framework for understanding how changes in lower-limb positioning modulate neuromuscular activation and force distribution. Therefore, the aim of this study is to comparatively examine the mechanobiological and neuromuscular effects of these foot-position techniques on muscle activation, joint angles, ground reaction forces, and postural balance parameters in both front and back squat variations. We hypothesized that changes in foot position would lead to distinct alterations in muscle activation patterns, joint kinematics, and kinetic outputs during both front and back squat exercises, with dorsiflexion-enhanced and flat-foot positions producing greater quadriceps activation and vertical force outputs compared to the plantar-flexion-enhanced condition.

## 2 Materials and methods

### 2.1 Research design

This study was conducted to examine the effects of three different foot-position techniques—the flat position, the dorsiflexion enhanced (heel-elevated) position, and the plantar-flexion enhanced (forefoot-elevated) position—on electromyography, kinematic, and kinetic outcomes during front and back squat variations. The research employed an experimental repeated-measures design in which each participant experienced all conditions, thereby reducing inter-individual variability. To minimize potential order effects and strengthen internal validity, the order of conditions was randomized and counterbalanced.

### 2.2 Participants

Twelve healthy, right-hand–dominant male athletes (age: 23.4 ± 3.8 years; training experience: 8.08 ± 2.7 years) with at least 3 years of resistance-training background and technical proficiency were included in the study. Inclusion criteria required participants to be capable of performing both front and back squat variations with proper technique and to have no active orthopedic injuries affecting the lower limbs, no history of surgery, and no systemic health issues. Descriptive characteristics and performance values of the participants are summarized in Table 1.

**TABLE 1:** Participant characteristics and squat performance values (mean ± SD).

Variable	Mean ± SD
Chronological age (years)	23.4 ± 3.8
Training experience (years)	8.1 ± 2.7
Back squat 70% 1RM (kg)	101.5 ± 13.2
Front squat 70% 1RM (kg)	75.3 ± 12.4
Back squat 100% 1RM (kg)	145.0 ± 18.8
Front squat 100% 1RM (kg)	107.5 ± 17.7
Heel-elevated back squat 70% 1RM (kg)	101.8 ± 15.5
Heel-elevated front squat 70% 1RM (kg)	70.3 ± 11.8
Heel-elevated back squat 100% 1RM (kg)	145.4 ± 22.1
Heel-elevated front squat 100% 1RM (kg)	100.4 ± 16.9
Forefoot-elevated back squat 70% 1RM (kg)	86.6 ± 15.2
Forefoot-elevated front squat 70% 1RM (kg)	63.3 ± 9.5
Forefoot-elevated back squat 100% 1RM (kg)	123.8 ± 21.7
Forefoot-elevated front squat 100% 1RM (kg)	90.4 ± 13.6

Participant characteristics and squat performance values are presented as mean ± standard deviation (SD). *1RM: one-repetition maximum*.

Lower-limb dominance was determined using the kick-a-ball test, in which the leg used to strike a ball is identified as the dominant limb (van Melick et al., 2017). All participants were confirmed to have right-leg dominance according to this procedure.

The sample size was calculated using G*Power 3.1.9.2 software (Faul et al., 2009), based on previous similar studies. With an assumed effect size of f = 0.50, a significance level of α = 0.05, and a statistical power of 1–β = 0.95, a minimum of 12 participants was determined to be sufficient. The study was conducted in accordance with the principles of the Declaration of Helsinki (World Medical Association, 2013) and was approved by the Near East University Scientific Research Evaluation and Ethics Committee (Approval Date: 11.03.2022; Approval No: YDU/2022/104-1564). Written informed consent was obtained from all participants prior to their inclusion in the study.

### 2.3 Instruments

For the collection of electromyographic (EMG) and physiological data, a portable, eight-channel, dual-mode data acquisition system (Myomonitor IV, Delsys Inc., Boston, MA, United States) was used. Data recording was carried out via EMG Works Acquisition 4.0.5 software provided by the same manufacturer. The system amplifier had a bandwidth of 20–450 Hz, operated with a 9 VDC power input, and featured an 80 dB common-mode rejection ratio. Signals were sampled at a rate of 1000 Hz, transmitted wirelessly to the host computer, and monitored and stored in real time.

Surface electrodes were placed on seven muscles of the participants' dominant (right) lower limb—vastus lateralis, vastus medialis, rectus femoris, semitendinosus, biceps femoris, gluteus maximus, and erector spinae (ES)—to record electrical activity, in accordance with established surface EMG measurement guidelines (Surface, 1999). To ensure optimal signal quality, the skin at the electrode sites was shaved and cleansed with alcohol to reduce impedance. The electrodes (DE-2.3, Delsys Inc.) were positioned along the longitudinal axis of each muscle in accordance with the procedures outlined in the SENIAM guidelines (Hermens et al., 1999). Each sensor consisted of two parallel silver bars (10 mm long, 1 mm diameter) with an inter-electrode distance of 10 mm. A common reference electrode was placed over the right iliac crest.

For kinematic analysis, reflective markers were placed on the following anatomical landmarks: the distal point of the right forefoot (fifth metatarsal head, aligned with the base of the little toe), lateral malleolus, superior edge of the lateral tibial plateau, greater trochanter of the femur, and the right end of the Olympic bar. Two-dimensional spatial reconstruction was performed using a calibration plane consisting of eight control points. The exercises were executed with a 20.5 kg Olympic barbell and a squat rack.

Kinetic data were collected using a Bertec 4060-NC force platform with a sampling frequency of 1000 Hz. The platform coordinate system followed the manufacturer's definition, where the X-axis corresponds to the medio-lateral direction, the Y-axis to the antero-posterior direction, and the Z-axis to the vertical direction. The recorded signals were processed through a 10 Hz low-pass Butterworth filter, and from these data, ground reaction forces (Fx: medio-lateral, Fy: antero-posterior, Fz: vertical), moments (Mx: sagittal-plane related, My: frontal-plane related, Mz: transverse-plane related), and center of pressure components (COPx: medio-lateral, COPy: antero-posterior) were calculated. Kinetic measurements were performed only on the dominant lower limb and through a single force platform, which represents one of the methodological limitations of the study. All systems were synchronized via DAQ4/IDAC, while camera–EMG synchronization was ensured using an LED triggering method.

### 2.4 Procedure

Participants took part in two separate sessions spaced 1 week apart. One week prior to the main testing session, a preliminary session was conducted to introduce the experimental protocol. In this session, all testing procedures were explained in detail, and participants were given the opportunity to ask questions. Additionally, one-repetition maximum (1RM) values for both the front squat and back squat variations were determined for each participant.

#### 2.4.1 One-repetition maximum (1RM) determination protocol

To determine the loads used in the study, the one-repetition maximum (1RM) testing protocol described by Kraemer et al., 1995 was applied. Participants first completed a general warm-up on a stationary cycle ergometer for 3–5 min, followed by preparatory sets consisting of 8–10 repetitions at approximately 50% of the estimated 1RM, 3–5 repetitions at 75% of the 1RM, and 1–3 repetitions at 90% of the 1RM. For each participant, 1RM values were determined separately for the front and back squat variations performed under the flat-foot, dorsiflexion-enhanced (heel-elevated), and plantar-flexion-enhanced (forefoot-elevated) positions. If a trial could not be completed with proper form, the load was not increased, and the attempt was repeated with strict attention to lifting technique and range of motion. Rest intervals of 3–5 min were provided between attempts.

Seventy percent of the previously determined 1RM was used as the working load during the experimental sessions for electromyography, kinematic, and kinetic data collection. This intensity was selected to promote moderate-to-high motor-unit recruitment and to reliably detect intermuscular activation differences (Gullett et al., 2009; Kraemer, 2004). After the preparatory stages, participants completed the 1RM test through successive trials with progressively increasing loads until proper lifting form, full range of motion, or technical proficiency could no longer be maintained. During each trial, the point at which the knee joint reached 90° of flexion was defined as the lower limit of the squat and was standardized using adjustable stoppers.

#### 2.4.2 Maximal voluntary isometric contraction (MVIC) measurements

Immediately after electrode placement, MVIC data were recorded from the quadriceps, hamstrings, gluteus maximus, and erector spinae muscle groups in accordance with the procedures recommended by Konrad (2005). For each muscle group, three separate MVIC trials lasting 3 s were performed in a randomized order, with 1-min rest intervals between trials.

#### 2.4.3 Test procedure

All participants performed two to three warm-up sets prior to testing. Exercises were carried out at 70% of the previously determined 1RM, and movements were initiated upon verbal command. To ensure consistent movement speed, all repetitions were performed using a metronome set at 60 beats per minute, with approximately 2 s allocated for the descent phase (full extension to 90° knee flexion) and 2 s for the ascent phase. Participants were not allowed to use a self-selected tempo and were required to follow the auditory timing cues throughout the testing. For both squat techniques, the starting and ending position was defined as full knee extension (180°). From this position, participants executed the descent phase by lowering until at least 90° of knee flexion was reached, and then returned to the initial position to complete the ascent phase.

During the back squat, the barbell was placed on the trapezius muscle; hips and knees were flexed until the thighs were parallel to the ground, while the back was kept straight, heels remained in contact with the floor, and knees were aligned with the feet throughout the movement. In the front squat, the barbell was positioned on the anterior deltoids and clavicles; elbows were lifted forward so that the upper arms were parallel to the ground, and the descent and ascent phases were performed similarly to the back squat. Rest intervals of 3–5 min were provided between all trials.

### 2.5 Data acquisition and analysis systems

#### 2.5.1 Electromyography (EMG)

Muscle activations were recorded using a portable eight-channel Myomonitor IV system (Delsys Inc., Boston, MA, United States). Raw electromyography signals were preprocessed using a fourth-order Butterworth band-pass filter (20–450 Hz) applied in a zero-phase (bidirectional) manner to prevent phase distortion, followed by a 50 Hz notch filter to remove power-line interference. The selected 20–450 Hz frequency range reflects standard biomechanical recommendations for surface electromyography processing, as frequencies below 20 Hz primarily contain motion artefacts and movement-related noise, while frequencies above 450 Hz represent non-physiological system noise (Winter, 2009). Although certain studies suggest that specific frequency bands may reflect different motor-unit firing characteristics, the present study focused on full-band electromyography amplitude, consistent with the primary aim of comparing overall muscle activation across foot-position conditions.

After filtering, signals were full-wave rectified and processed with a 100 m RMS window (8% overlap). Electrodes were placed on the dominant (right) leg over the vastus lateralis (VL), vastus medialis (VM), rectus femoris (RF), gluteus maximus (GM), semitendinosus (ST), biceps femoris (BF), and erector spinae (ES) muscles in accordance with SENIAM guidelines (Hermens et al., 1999). A reference electrode was positioned over the right iliac crest.

#### 2.5.2 Kinematics

Kinematic data were captured using a digital camera operating at 100 Hz in the sagittal plane. Reflective markers were placed on anatomical landmarks including the fifth metatarsal head, lateral malleolus, lateral tibial plateau, greater trochanter, and the end of the Olympic bar. A calibration frame consisting of eight control points was used for spatial scaling. Data were digitized and processed in SaSuite 2D software using a 6–12 Hz low-pass Butterworth filter.

#### 2.5.3 Kinetics

Ground reaction forces and moment variables were recorded using a Bertec 4060-NC force platform sampled at 1000 Hz. Signals were filtered using a 10 Hz low-pass Butterworth filter, and force components (Fx, Fy, Fz), moments (Mx, My, Mz), and center of pressure trajectories (COPx, COPy) were extracted. Due to the use of a single platform, kinetic variables were calculated for the dominant limb.

#### 2.5.4 Synchronization

All measurement systems used in this study included the Delsys Trigno Wireless electromyography system, a high-speed video camera operating at a shutter speed of 1/250 s, and AMCap software (Microsoft, Version 3.0.9) for video capture. Synchronization across electromyography and video recordings was achieved using a National Instruments USB-6501 digital I/O interface, which provided a precise LED-based triggering signal to align the onset of both modalities. This configuration ensured accurate temporal matching of kinematic and neuromuscular data during each squat trial.

### 2.6 Data processing

#### 2.6.1 Electromyography processing

Raw electromyography signals were segmented into descent and ascent phases, filtered, rectified, and integrated. Mean electromyography amplitudes were normalized to each participant’s highest MVIC value. Trials with artifacts or signal loss were excluded prior to analysis.

#### 2.6.2 Kinematic processing

Video segments corresponding to each repetition were cropped and digitized. Joint coordinate data were smoothed using a moving average filter, and ankle, knee, and hip angles were computed separately for descent and ascent.

#### 2.6.3 Kinetic processing

Force-time and moment-time data were segmented by movement phases and summarized at the participant level. Mean values were derived for each condition and used in subsequent statistical analyses.

### 2.7 Variables

The independent variable was the foot-position technique used during the squat: flat-foot, dorsiflexion-enhanced (heel-elevated), and plantar-flexion-enhanced (forefoot-elevated). Foot positions were standardized using 5-cm solid hardwood blocks, which provided a fully rigid, non-compressible surface during all squat conditions. All trials were performed barefoot to avoid proprioceptive differences caused by footwear.

Dependent variables consisted of:

1. Electromyography outcomes: normalized RMS values for VL, VM, RF, GM, ST, BF, and ES.
2. Kinematic outcomes: ankle, knee, and hip joint angles for descent and ascent.
3. Kinetic outcomes: ground reaction forces (Fx, Fy, Fz), joint moments (Mx, My, Mz), and center of pressure trajectories (COPx, COPy).

### 2.8 Data analysis

Kinematic, kinetic, and electromyography data were analyzed using repeated-measures analysis of variance (rm-ANOVA) with the factors squat technique × squat variation (front/back) × phase (descent/ascent). Homogeneity of variances and sphericity were tested using the Shapiro–Wilk and Mauchly tests, respectively. When necessary, the Greenhouse–Geisser correction was applied. Bonferroni-adjusted *post hoc* tests were conducted to identify the sources of significant differences. Considering three pairwise comparisons, the Bonferroni-corrected significance threshold was set at p < 0.017 (0.05/3). Effect sizes were reported as partial eta squared (η^2^) and interpreted according to Cohen (1988) thresholds (small ≥0.01, medium ≥0.06, large ≥0.14). In addition, Cohen’s d values were calculated for all pairwise *post hoc* comparisons (flat vs. heel-elevated, flat vs. forefoot-elevated, and heel-elevated vs. forefoot-elevated) using pooled standard deviations to quantify the magnitude of differences between techniques. The overall level of statistical significance was set at p < 0.05.

## 3 Results

### 3.1 Electromyography (EMG)

Significant differences in electromyography activation levels were observed across muscles depending on foot-position technique and squat phase. Among the quadriceps, the vastus lateralis (VL) and vastus medialis (VM) showed the greatest sensitivity to foot positioning, with significantly higher activations in the flat-foot and heel-elevated techniques compared with the forefoot-elevated technique during the back squat ascent (VL: F (2,11) = 12.75, p < 0.001, ηp^2^ = 0.54; VM: F (2,11) = 16.65, p < 0.001, ηp^2^ = 0.60; large effects). The rectus femoris (RF) also displayed significant differences, with greater activation in the flat-foot and heel-elevated positions during both the back squat ascent (F (2,11) = 11.72, p < 0.001, ηp^2^ = 0.52; large effect) and the front squat descent phases (F (2,11) = 6.06, p = 0.008, ηp^2^ = 0.36; large effect). For the posterior chain, the gluteus maximus (GM) exhibited higher activation in the flat-foot and heel-elevated techniques during the back squat ascent (F (2,11) = 7.22, p = 0.004, ηp^2^ = 0.40; large effect), while the erector spinae (ES) differed only during the front squat descent, with greater activation in the flat-foot condition compared to the heel-elevated condition (F (2,11) = 4.60, p = 0.021, ηp^2^ = 0.30; large effect). No significant differences were found in semitendinosus (ST) or biceps femoris (BF) activations (p > 0.05, ηp^2^ ≤ 0.22; small-to-medium effects). Detailed electromyography values for each muscle and phase are presented in Table 2.

**TABLE 2:** Electromyography (EMG) activity of all muscles across squat phases and foot-position techniques (mean ± SD, normalized to MVIC).

Muscle	Phase	FHmean ± SD	HEmean ± SD	FEmean ± SD	F (2,11)	p	ηp^2^	Post-hoc differences
Vastus lateralis	Back ascent	60.73 ± 18.51	51.10 ± 16.11	39.96 ± 16.50	12.75	<0.001*	0.54	a–b, a–c, b–c
	Back descent	50.12 ± 20.44	49.36 ± 21.46	40.08 ± 20.78	2.44	0.110	0.18	ns
Vastus medialis	Back ascent	61.17 ± 24.14	58.35 ± 23.33	36.90 ± 15.85	16.65	<0.001*	0.60	a–c, b–c
	Back descent	53.02 ± 19.38	51.19 ± 21.52	39.61 ± 15.86	7.61	0.003*	0.41	a–c, b–c
Rectus femoris	Back ascent	22.27 ± 10.13	23.77 ± 10.20	13.32 ± 5.45	11.72	<0.001*	0.52	a–c, b–c
	Front descent	19.83 ± 6.66	19.57 ± 5.59	14.39 ± 5.48	6.06	0.008*	0.36	a–c, b–c
Gluteus maximus	Back ascent	22.27 ± 10.13	23.77 ± 10.20	13.32 ± 5.45	7.22	0.004*	0.40	a–c, b–c
Erector spinae	Front descent	54.09 ± 23.79	37.72 ± 15.42	42.75 ± 13.64	4.60	0.021*	0.30	a–b
Semitendinosus	All phases	–	–	–	ns	>0.05	<0.14	ns
Biceps femoris	Back ascent	19.75 ± 10.15	16.98 ± 11.16	12.69 ± 7.86	3.04	0.068	0.22	ns

Values are presented as mean ± standard deviation and normalized to MVIC (maximum voluntary isometric contraction).

FH, flat heel; HE, Heel-Elevated; FE, Forefoot-Elevated. ns = non-significant; *p < .05; ηp^2^= partial eta squared (effect size).

### 3.2 Kinematic joint angles

Significant differences were observed across all four phases, with dorsiflexion angle being lowest in the forefoot-elevated (FE) condition and highest in the heel-elevated (HE) condition. The finding that heel-elevated expands the kinematic window of motion is consistent with previous literature (Macrum et al., 2012; Charlton et al., 2017; Duan et al., 2025). Similarly, Lu et al. (2022) reported that acute heel-elevated produced significant improvements in both squat depth and ankle dorsiflexion.

The forefoot-elevated (FE) technique produced greater knee flexion across most phases of both front and back squats (F (2,11) = 9.94, p = 0.001, ηp^2^ = 0.48; large effect for back ascent; F (2,11) = 6.98, p = 0.005, ηp^2^ = 0.39; large effect for front descent). This outcome can be attributed to the anterior shift of the center of mass and the resulting increase in knee extensor moments (Lorenzetti et al., 2018; Hartmann et al., 2013; Mauntel et al., 2013; Escamilla et al., 2001). Supporting this interpretation, Yavuz et al. (2015) demonstrated that knee flexion angles are strongly influenced by technique. These findings reinforce the present results, suggesting that foot position manipulations can substantially affect knee kinematics.

Specifically, while the forefoot-elevated position maintains the knees in a more extended posture and shifts loading demands from the hip to the knee, both flat-foot (FF) and heel-elevated (HE) techniques permit greater flexion, thereby enhancing quadriceps activation (Cotter et al., 2013).

Hip joint angles did not differ significantly across techniques (ηp^2^ ≤ 0.15; medium effects). This suggests that hip kinematics remain relatively stable regardless of foot positioning, with depth, trunk inclination, and loading conditions exerting a more decisive influence (Larsen et al., 2021; Lorenzetti et al., 2018; List et al., 2013; Schoenfeld, 2010b). Although greater hip flexion might theoretically be expected in the forefoot-elevated (FE) position, the more upright trunk orientation observed in this technique may have counterbalanced such an effect (Glassbrook et al., 2017).

Overall, the kinematic findings revealed large effect-size differences primarily in the ankle (ηp^2^ = 0.34–0.51; large effects) and knee (ηp^2^ = 0.28–0.48; large effects) joint angles. These alterations not only influenced joint mechanics but also shaped muscle activation profiles. For instance, the more upright trunk position in the forefoot-elevated technique was associated with reduced activation of posterior chain muscles such as the erector spinae (ES) and gluteus maximus (GM), alongside relatively diminished quadriceps loading. From a kinematic perspective, these results indicate that squat mechanics are influenced not only by loading intensity but also by technical variations (Monteiro et al., 2022; Goršič et al., 2024). Therefore, the choice of squat technique should incorporate a holistic consideration of individual mobility levels and target muscle activation outcomes. Detailed joint-angle values for the ankle, knee, and hip during front and back squats are presented in Table 3.

**TABLE 3:** Joint angles (°) during front and back squats across foot-position techniques (mean ± SD, normalized to movement phase).

Joint	Phase	FH mean ± SD	HE mean ± SD	FE mean ± SD	F (2,11)	p	ηp^2^	Post-hoc differences
Ankle	Back descent	112.97 ± 7.25	115.56 ± 6.18	107.04 ± 6.79	11.43	<0.001*	0.51	a–c, b–c
	Back ascent	115.62 ± 7.10	118.05 ± 4.16	112.31 ± 4.38	6.77	0.005*	0.38	a–c, b–c
	Front descent	110.62 ± 6.46	114.58 ± 5.78	106.88 ± 7.12	11.06	<0.001*	0.50	a–c, b–c
	Front ascent	115.72 ± 6.29	116.71 ± 6.88	110.61 ± 8.43	5.69	0.010*	0.34	a–c, b–c
Knee	Back descent	141.64 ± 6.94	139.81 ± 5.07	142.40 ± 6.42	1.19	0.324	0.10	ns
	Back ascent	142.27 ± 5.87	141.27 ± 5.35	151.62 ± 10.09	9.94	0.001*	0.48	a–c, b–c
	Front descent	137.52 ± 6.96	136.39 ± 5.99	142.87 ± 5.98	6.98	0.005*	0.39	a–c, b–c
	Front ascent	140.35 ± 10.67	139.36 ± 9.36	147.52 ± 8.33	4.36	0.025*	0.28	a–c, b–c
Hip	Back descent	147.89 ± 6.29	149.45 ± 6.00	145.13 ± 7.59	1.87	0.178	0.15	ns
	Back ascent	145.42 ± 6.35	149.45 ± 6.00	151.48 ± 12.51	1.92	0.170	0.15	ns
	Front descent	144.20 ± 10.48	145.31 ± 9.45	143.73 ± 7.63	0.22	0.804	0.02	ns
	Front ascent	141.79 ± 15.34	144.00 ± 9.10	144.14 ± 11.12	0.22	0.808	0.02	ns

Values are presented as mean ± standard deviation and normalized to movement phase. See Table 2 for abbreviation definitions. ns, non-significant; *p < .05.

### 3.3 Ground reaction force components (FX, FY, FZ)

Significant differences were observed in the ground reaction force (GRF) parameters depending on the foot position technique and squat phase.

For the medio–lateral (FX) component, a significant difference emerged only during the front squat descent phase (F(2,11) = 4.99, p = 0.016, ηp^2^ = 0.31; large effect). Post-hoc analyses indicated that all pairwise comparisons (FH–HE, FH–FE, and HE–FE) differed significantly, with the forefoot-elevated (FE) technique showing higher medio–lateral force fluctuations. In contrast, no significant differences were found in the back squat phases (p > 0.05, ηp^2^ < 0.10; small effects).

Regarding the antero–posterior (FY) component, a significant difference was found only in the front squat descent phase (F(2,11) = 4.01, p = 0.033, ηp^2^ = 0.27; large effect), where the FE technique produced greater forward–backward force shifts than the flat-heel (FH) condition. No significant differences were detected in other phases (p > 0.05, ηp^2^ ≤ 0.16; small-to-medium effects).

For the vertical (FZ) component, a clear and consistent pattern emerged across both squat variations. The FE technique produced significantly lower vertical forces than both FH and HE during the back squat descent (F(2,11) = 11.58, p<0.001, – = 0.51; large effect), back squat ascent (F(2,11) = 11.59, p<0.001, – = 0.51; large effect), and front squat descent (F(2,11) = 4.17, p = 0.029, ηp^2^ = 0.28; large effect) phases. This indicates that elevating the forefoot reduced the capacity to generate downward and propulsive vertical forces.

Collectively, these findings suggest that heel elevation enhances both stability and vertical force production, whereas forefoot elevation increases antero-posterior and medio-lateral instability, particularly during the descent phase. Detailed ground reaction force values across all axes are presented in Table 4.

**TABLE 4:** Ground reaction force components (Fx, Fy, Fz) during front and back squats across foot-position techniques (mean ± SD, normalized to body weight).

Axis	Phase	FH mean ± SD	HE mean ± SD	FE mean ± SD	F (2,11)	p	ηp^2^	Post-hoc differences
FY (A–P)	Back descent	5.28 ± 49.41	−10.31 ± 43.23	3.07 ± 30.52	0.52	0.605	0.05	ns
	Back ascent	19.20 ± 46.92	17.56 ± 33.70	−4.85 ± 57.20	0.85	0.441	0.07	ns
	Front descent	−41.52 ± 57.86	−9.45 ± 40.87	27.14 ± 56.03	4.99	0.016*	0.31	a–b, a–c, b–c
	Front ascent	−45.04 ± 63.92	−2.30 ± 52.29	16.53 ± 61.72	3.24	0.058	0.23	ns
FY (A–P)	Back descent	6.57 ± 38.27	−4.59 ± 40.20	−3.85 ± 30.89	0.39	0.680	0.04	ns
	Back ascent	8.40 ± 33.53	7.02 ± 26.19	−23.83 ± 66.58	1.73	0.200	0.14	ns
	Front descent	−52.46 ± 69.40	−17.29 ± 52.25	11.57 ± 50.35	4.01	0.033*	0.27	a–c
	Front ascent	−44.08 ± 51.62	−8.02 ± 55.02	−2.78 ± 63.14	2.07	0.151	0.16	ns
FZ (vertical)	Back descent	1732.09 ± 204.45	1776.44 ± 235.77	1578.79 ± 208.63	11.58	<0.001*	0.51	a–c, b–c
	Back ascent	1770.10 ± 188.95	1740.02 ± 251.02	1544.50 ± 204.10	11.59	<0.001*	0.51	a–c, b–c
	Front descent	1496.13 ± 174.63	1505.44 ± 160.98	1394.01 ± 146.30	4.17	0.029*	0.28	a–c, b–c
	Front ascent	1491.93 ± 222.73	1476.99 ± 135.46	1375.81 ± 150.05	3.09	0.066	0.22	ns

Values are presented as mean ± standard deviation and normalized to body weight. See Table 2 for abbreviation definitions. ns, non-significant; *p < .05.

### 3.4 Rotational moment components (Mx, My, Mz)

Analysis of the rotational moment components revealed limited but noteworthy variations across foot positions. For the X-axis (Mx), which reflects sagittal-plane rotational torque, no statistically significant differences were detected in any phase (p > 0.05), although values during the back squat descent (F(2,11) = 3.32, p = 0.055, ηp^2^ = 0.23) and front squat descent (F(2,11) = 3.33, p = 0.055, ηp^2^ = 0.23) approached significance, suggesting only a moderate influence of foot positioning on sagittal-plane torque generation.

Similarly, Y-axis (My) values, representing frontal-plane rotational moments, did not differ significantly across phases (p > 0.05, ηp^2^ = 0.05–0.16), indicating that altering foot elevation did not substantially affect frontal-plane moment distribution.

In contrast, a significant effect emerged for the Z-axis (Mz) during the back squat descent phase (F(2,11) = 6.19, p = 0.007, ηp^2^ = 0.36), with the forefoot-elevated technique producing lower Mz values than the flat-foot and heel-elevated techniques, implying reduced transverse-plane rotational stability in this condition. Other phases showed no significant differences (p > 0.05, ηp^2^ ≤ 0.24). Overall, these findings suggest that while foot positioning exerted minimal influence on sagittal (Mx) and frontal (My) rotational moments, it played a more pronounced role in transverse-plane rotation, particularly during the back squat descent. Detailed rotational moment values for all axes are presented in Table 5.

**TABLE 5:** Rotational moment components (Mx, My, Mz) during front and back squats across foot-position techniques (mean ± SD, normalized to body weight).

Axis	Phase	FH mean ± SD	HE mean ± SD	FE mean ± SD	F (2,11)	p	ηp^2^	Post-hoc differences
Mx	Back descent	3.04 ± 27.74	0.72 ± 28.83	−15.91 ± 25.93	3.32	0.055	0.23	ns
	Back ascent	−5.05 ± 40.30	−22.26 ± 28.65	−14.89 ± 24.88	0.95	0.402	0.08	ns
	Front descent	16.02 ± 26.71	4.55 ± 28.37	−6.90 ± 17.32	3.33	0.055	0.23	ns
	Front ascent	24.11 ± 34.32	5.82 ± 31.32	−8.14 ± 24.66	3.01	0.070	0.22	ns
My	Back descent	12.63 ± 42.55	12.58 ± 61.18	−5.84 ± 27.40	0.75	0.483	0.06	ns
	Back ascent	12.21 ± 40.76	8.96 ± 55.68	−21.83 ± 34.98	2.07	0.151	0.16	ns
	Front descent	−6.44 ± 36.46	−0.81 ± 31.51	13.09 ± 40.74	0.90	0.423	0.08	ns
	Front ascent	−7.48 ± 41.34	9.27 ± 36.50	8.93 ± 50.60	0.61	0.550	0.05	ns
Mz	Back descent	6.57 ± 38.27	−4.59 ± 40.20	−3.85 ± 30.89	6.19	0.007*	0.36	a–c, b–c
	Back ascent	8.40 ± 33.53	7.02 ± 26.19	−23.83 ± 66.58	3.39	0.052	0.24	ns
	Front descent	−52.46 ± 69.40	−17.29 ± 52.25	11.57 ± 50.35	1.63	0.218	0.13	ns
	Front ascent	−44.08 ± 51.62	−8.02 ± 55.02	−2.78 ± 63.14	0.20	0.819	0.02	ns

Values are presented as mean ± standard deviation and normalized to body weight. See Table 2 for abbreviation definitions. ns = non-significant; *p < .05.

### 3.5 Center of pressure (COPx, COPy)

Analysis of center of pressure displacement revealed no statistically significant differences among foot positions in either the medio–lateral (COPx) or antero–posterior (COPy) directions (p > 0.05).

For COPx, none of the phases showed significance, although small-to-medium effect sizes (ηp^2^ = 0.05–0.16) were observed, particularly during the back squat ascent phase (F(2,11) = 2.07, p = 0.150, ηp^2^ = 0.16), indicating only minor medio–lateral adjustments in pressure trajectory with foot elevation.

COPy results similarly showed no significant differences across phases, with the largest deviation occurring during the front squat descent (F(2,11) = 3.29, p = 0.056, ηp^2^ = 0.23), approaching significance and suggesting a possible trend toward increased antero–posterior sway in the forefoot-elevated condition.

Overall, these results indicate that neither heel nor forefoot elevation meaningfully altered balance control, and the minor variations observed in COPx and COPy likely reflect subtle compensatory adjustments rather than impactful destabilization. Detailed COP displacement values are presented in Table 6.

**TABLE 6:** Center of pressure displacement (COPx, COPy) during front and back squats across foot-position techniques (mean ± SD, normalized to stance width).

Direction	Phase	FH mean ± SD	HE mean ± SD	FE mean ± SD	F (2,11)	p	*ηp* ^*2*^	Post-hoc differences
COPx (M–L)	Back descent	−0.01 ± 0.03	0.00 ± 0.04	0.00 ± 0.02	0.65	0.533	0.06	ns
	Back ascent	−0.01 ± 0.02	0.00 ± 0.03	0.01 ± 0.02	2.07	0.150	0.16	ns
	Front descent	0.01 ± 0.02	0.00 ± 0.02	−0.01 ± 0.03	1.08	0.356	0.09	ns
	Front ascent	0.01 ± 0.03	0.00 ± 0.02	0.00 ± 0.04	0.53	0.593	0.05	ns
COPy (A–P)	Back descent	0.00 ± 0.02	0.00 ± 0.02	0.00 ± 0.02	0.09	0.911	0.01	ns
	Back ascent	0.00 ± 0.02	−0.01 ± 0.02	−0.01 ± 0.02	0.74	0.489	0.06	ns
	Front descent	0.01 ± 0.02	0.00 ± 0.02	0.00 ± 0.01	3.29	0.056	0.23	ns
	Front ascent	0.01 ± 0.02	0.00 ± 0.02	−0.01 ± 0.02	2.64	0.094	0.19	ns

Values are presented as mean ± standard deviation and normalized to stance width. See Table 3 for abbreviation definitions. ns, non-significant.

## 4 Discussion

The present study demonstrated that manipulating foot position substantially alters neuromuscular activation, joint kinematics, ground reaction forces and, to a lesser degree, postural stability during both front and back squat variations. Overall, heel-elevated (HE) and flat-foot squatting produced higher quadriceps activation and greater vertical force outputs, whereas forefoot-elevated (FE) generally limited knee flexion and reduced the mechanical demand placed on both the quadriceps and posterior chain. These findings align with previous work showing that squat technique modifications can markedly influence load distribution, depth, and movement efficiency (Schoenfeld, 2010a; Escamilla et al., 2001; Saeterbakken et al., 2017). Prior studies have similarly demonstrated that altering foot wedges leads to meaningful shifts in lower-extremity muscle activation during loaded tasks, particularly affecting thigh and hamstring activity (Ghasemi, Anbarian and Esmaeili, 2018). From a mechanobiological perspective, changes in foot mechanics appear to modify how external loads are transmitted through the kinetic chain, thereby shaping neuromuscular recruitment patterns (Wackerhage et al., 2019; Ghasemi and Anbarian, 2020; Stone et al., 2024).

Regarding muscle activation, the quadriceps muscles—particularly the vastus lateralis (VL) and vastus medialis (VM)—showed the greatest sensitivity to foot-position changes. Both VL and VM exhibited significantly higher activation in the flat-foot (FH) and heel-elevated (HE) techniques compared with the forefoot-elevated (FE) condition, especially during the ascent phase of the back squat. These results are consistent with evidence indicating that increased dorsiflexion improves knee extensor moment arms and enhances quadriceps recruitment (Charlton et al., 2017; da Costa et al., 2021). Recently published meta-analyses further support this interpretation, demonstrating that altering heel height or wedge configuration systematically increases thigh muscle activation during squatting and gait (Ghasemi, Gholami-Borujeni and Briem, 2024; Ghasemi, Gholami-Borujeni and Babagoltabar-Samakoush, 2025). Posterior-chain muscles such as the gluteus maximus (GM) and erector spinae (ES) were also affected, with greater ES activation occurring in the flat-foot condition due to increased trunk inclination (Larsen et al., 2021). In contrast, the hamstrings showed no significant changes, reflecting their stabilizing rather than primary agonist function during bilateral squats (Kubo et al., 2019; McBride et al., 2002).

Kinematic outcomes highlighted that ankle and knee joint angles responded most strongly to foot-position manipulation. Heel-elevated significantly increased dorsiflexion range, enabling deeper squatting and improved overall kinematic efficiency—an effect consistent with prior reports linking ankle mobility to increased squat depth (Macrum et al., 2012; Lu et al., 2022; Duan et al., 2025). Conversely, the forefoot-elevated technique shifted the center of mass anteriorly, resulting in greater knee flexion demands and modified extensor moment profiles (Lorenzetti et al., 2018; Hartmann et al., 2013). Hip joint angles remained largely unaffected by foot position, confirming that hip kinematics are more strongly influenced by squat depth and loading conditions than by minor alterations beneath the foot (List et al., 2013; Schoenfeld, 2010b). These patterns are consistent with recent findings showing that technique-related factors—including bar placement and squat variation—can meaningfully alter trunk inclination and lower-limb joint angles (Goršič et al., 2024). Importantly, the kinematic modifications observed here directly parallel the muscle activation findings: positions that facilitate greater knee flexion (FH, heel-elevated) generally produced higher quadriceps activation, while positions promoting a more upright trunk (forefoot-elevated) reduced posterior-chain involvement.

Kinetic outcomes further demonstrated the impact of foot placement on mechanical demands. Vertical ground reaction forces (Fz) were significantly higher in the flat-foot and heel-elevated positions, reflecting their mechanical advantage for vertical force transmission, while the forefoot-elevated condition consistently produced lower vertical outputs. These results align with previous studies showing that deeper squats and improved ankle mobility enhance vertical force production (Seitz et al., 2014; Duan et al., 2025). Although frontal-plane (Fy) and antero–posterior (Fx) forces showed only condition-specific differences, similar mediolateral and anteroposterior loading alterations have been reported when stance or foot mechanics are modified, such as in changes to stance width (Paoli et al., 2009). Transverse-plane rotational moments (Mz) were notably lower in the forefoot-elevated position, indicating reduced rotational stability and limited resistance to transverse-plane shear forces. This agrees with prior investigations showing that squat technique modifications can meaningfully affect rotational torque requirements (Swinton et al., 2012), particularly when foot mechanics alter center-of-mass control strategies. Moreover, previous biomechanical comparisons of squat variations have demonstrated that even small technical adjustments—such as bar position changes—can significantly modify moment profiles around critical phases like the sticking region (Kristiansen et al., 2021), supporting the interpretation that foot-position changes are sufficient to alter kinetic demands.

Postural stability, as measured through center-of-pressure (COP) displacement, displayed no statistically significant differences among foot positions, although small-to-medium effect sizes suggested subtle adaptations. The forefoot-elevated condition showed a trend toward greater antero–posterior adjustments, consistent with its forward weight-shift characteristics (Ottinger et al., 2023). However, the overall stability across all conditions reflects the inherent robustness of bilateral squatting, where symmetrical loading and a wide base of support minimize destabilizing forces (Saeterbakken and Fimland, 2013; Andersen et al., 2014). This is further supported by evidence that even minor changes in base-of-support width can alter postural control strategies and foot-loading symmetry during squatting (Kędziorek and Błażkiewicz, 2022). Individual factors such as ankle mobility, neuromuscular coordination, and training experience likely played a greater role in COP behaviors than foot position alone (Lorenzetti et al., 2018; Ishida et al., 2022).

From a mechanobiological standpoint, these findings collectively demonstrate that small adjustments in foot position can substantially influence how mechanical loads propagate through the lower-limb kinetic chain. This supports established mechanotransduction models where mechanical stimuli—such as altered joint angles, center-of-mass shifts, and changes in torque—produce corresponding adaptations in muscle activation and movement strategy (Ingber, 2006; Murach and Bagley, 2016). The current study reinforces the idea that modifying heel height or forefoot-elevated can be strategically employed to target specific muscle groups, optimize joint loading, and facilitate desired neuromuscular adaptations in both athletic performance and rehabilitation contexts (Wackerhage et al., 2019; Damas et al., 2019).

## 5 Conclusion

The present study shows that altering foot position meaningfully changes squat mechanics, particularly by modifying quadriceps activation, ankle kinematics, and vertical force production, while hip motion, frontal-plane loading, and overall postural stability remain largely unaffected. Heel-elevated and flat-foot squatting consistently enhanced neuromuscular and mechanical efficiency compared with forefoot-elevated. These findings suggest that simple adjustments at the foot–ground interface can be strategically used to target specific muscle groups, improve movement depth, and optimize load distribution. Such insights hold practical relevance for strength training, injury prevention, and rehabilitation programs, where foot-position modifications may serve as an effective and accessible tool for tailoring squat performance.

### 5.1 Limitations

Several limitations of the present study should be acknowledged. First, only young, highly trained male athletes were included. Because females typically demonstrate greater hip flexion, knee valgus, and different neuromuscular strategies during squatting (Kerrigan et al., 2000), the findings may not generalize to female populations or to older adults, who exhibit age-related changes in muscle activation and joint mechanics. Second, all participants had more than 8 years of resistance-training experience, which likely reflects neuromuscular adaptations not representative of novice lifters or the general population. Third, body composition variables such as BMI were not considered, although differences in body mass and fat distribution can influence joint loading and stabilization demands during squatting. Additionally, limb dominance was assessed using the kick-a-ball test to determine the dominant leg, but lower-limb dominance does not always parallel hand dominance and may contribute to asymmetrical loading patterns. Measuring only the dominant limb may therefore have influenced electromyography activity, kinematic variables, and ground reaction forces. Future studies should consider bilateral measurements, include participants with diverse demographics and training backgrounds, and incorporate body composition assessments to enhance generalizability and reduce potential bias.

### 5.2 Practical applications

The present findings indicate that foot-position modifications can be used strategically to tailor squat technique for training or rehabilitation needs. Heel-elevated enhances dorsiflexion, increases squat depth, and substantially boosts quadriceps activation, making it suitable for strength development or mobility-limited individuals. Flat-foot squatting provides balanced quadriceps and posterior-chain engagement and may be preferable for general lower-limb strengthening. Forefoot-elevated reduces quadriceps demand and vertical loading, which may benefit individuals with knee sensitivity, though increased knee flexion requires cautious load management. As balance measures remained largely stable across techniques, all three variations can be safely integrated into performance and rehabilitation settings.

### 5.3 Future research directions

Future studies should employ larger and more diverse samples that include both sexes and multiple performance levels. The application of three-dimensional kinematics and musculoskeletal modeling may offer deeper insights into joint moments and loading distributions. Additionally, multifactorial experimental designs incorporating variables such as bar placement, stance width, and load intensity, as well as longitudinal analyses addressing motor learning, fatigue, and injury risk indicators, are warranted.

Finally, the integration of portable electromyography systems and artificial intelligence–assisted analytic methods could facilitate the development of individualized, field-based squat protocols, thereby advancing both scientific understanding and applied practice.

## Data Availability

The raw data supporting the conclusions of this article will be made available by the authors, without undue reservation.
